# Adult ADHD predicts intimate partner violence perpetration and victimization irrespective of gender and age

**DOI:** 10.1038/s41598-024-74222-w

**Published:** 2025-02-10

**Authors:** Johannes Merscher, Steffen Barra, Anika May Xander, Paula Gratiela Patrasc, Petra Retz-Junginger, Wolfgang Retz

**Affiliations:** 1 Institute for Forensic Psychology and Psychiatry, Saarland University Medical Center, Homburg, Germany; 2 Department of Psychiatry and Psychotherapy, University Medical Center of the Johannes Gutenberg-University, Mainz, Germany

**Keywords:** Social behaviour, Human behaviour

## Abstract

Understanding the determinants of intimate partner violence (IPV) from perpetrator and victim perspectives has become a major objective of behavioral science. Empirical evidence suggests that adults at risk for Attention-Deficit/Hyperactivity Disorder (ADHD), compared to the general population, tend to have more conflictual partnerships, and the presence of ADHD increases the risk of aggressive behavior. Possible influences of gender have not been sufficiently investigated yet. Using data from an anonymous online survey, this study examined the relationship between ADHD and IPV in 316 male and female individuals with (n = 131) and without (n = 185) ADHD. Multiple linear regression analyses showed that adults at risk for ADHD had more frequently become both victims and perpetrators of IPV compared to the healthy control group. ADHD achieved significant incremental variance over gender and age. Thus, the presence of ADHD seems to be an important risk factor for IPV irrespective of gender and age. Accordingly, research and treatment approaches focusing on ADHD must not neglect the risk of IPV among patients but should offer specific psychological support.

## Introduction

Attention-Deficit/Hyperactivity Disorder (ADHD) is characterized by continuous attention deficits, hyperactivity and/or impulsivity patterns that lead to a wide range of functional impairments in academic, occupational, or social domains^1^.

On account of the considerable heritability of about 70%^2^ and the high prevalence between 8 and 12% worldwide^3^, ADHD has been considered as the most common psychiatric disorder in childhood and adolescence. However, the high symptomatic persistence into adulthood^4,5^ drew attention to the impact of ADHD throughout adulthood and, respectively, to related psychosocial consequences in a wide range of inter- and intrapersonal domains^6,7^.

Prior research has identified the comorbid occurrence of ADHD and aggression^8^ and stated ADHD as a risk factor for delinquent behavior^9,10^. Taken together, ADHD symptoms are not only associated with an increased number of violent crimes and higher incarceration rates but also with an earlier onset of disruptive behavior and an increased risk for criminal recidivism and early relapse^9,11–13^.

Importantly, high impulsivity as a core symptom of ADHD increases the likelihood of disruptive and risky behaviors^14–18^. Moreover, poor emotional self-regulation and low self-control, which is a typical psychopathological feature associated with adult ADHD^19,20^, may additionally increase the risk for aggressive behavior towards others^21^.

Intimate partner violence (IPV) is a special form of violent behavior that refers to all forms of violence between intimate partners and can be understood from a dynamic developmental systems perspective in which couple aggression is conceptualized as an interactional pattern^22,23^. According to the criminal statistical data on IPV in Germany, more than 143,000 citizens were victims of IPV in 2021, with physical injury being the most commonly reported type of violence^24^. More than 80% of the victims were women, whereas approximately 80% of offenders were men. IPV is a socially widespread phenomenon independent of socioeconomic status or ethnic group. All forms of violence (physical, psychological or sexual) represent an immense stressor for IPV victims that sparks various noxious effects or traumatic consequences^25^. Numerous theories have tried to explain the emergence of IPV^26,27^ and each of them proposed multifactorial conditions for its occurrence. Hierarchical gender relations, abusive or negligent experiences in the offender’s past, the lack of promoting relationship abilities, and the victim’s psychological health are factors often discussed in the context of IPV^22^. These broad sociological theories include strain theory, which posits that societal structures and relationships create frustrations that lead some individuals to resort to violence; social disorganization theory, which suggests that neighbourhood environmental factors create social conditions that are conducive to criminal behaviour, including violence; and benefit theory, which posits that violence occurs when social costs are minimal and the benefits of violent acts outweigh the costs^28^. The extent to which these theories have effectively influenced interventions for the prevention and treatment of IPV remains limited^29^. Theories of IPV should take a more comprehensive approach, encompassing the perspectives of both victims and perpetrators, and incorporating evidence from a range of academic disciplines^26^.

Previous research has investigated the potential association between ADHD and IPV^30,31^. For instance, adults with more severe ADHD reported more negative couple functioning^32^.

According to prior findings, adults with childhood ADHD who were experiencing greater current ADHD symptom severity stated both higher rates of IPV perpetration and victimization when comparing them to adults without any ADHD history^30,33,34^ or to adults with ADHD histories but low current symptomatology^34^.

Regarding various types of violent behavior, preliminary evidence has shown that individuals with ADHD exhibit higher levels of psychological aggression and physical assault than controls in both perpetration and victimization^34^. Additionally, a differentiated inspection of retrospectively reported childhood ADHD symptoms has identified predictive links between inattention and violence perpetration without injury and between hyperactivity/impulsivity and violence perpetration resulting in injury^31^. Taken together, these findings stress the considerable impact of ADHD on elevated rates of IPV direction (perpetration and victimization) and psychological aggression as well as physical assault.

However, the published literature on the link between ADHD and IPV is somewhat discordant^35^ and limited in terms of the age of the subjects and possible gender differences. Although numerous studies considered aggressive behavior within intimate relationships of young adults with ADHD^30,33,36^, only a few studies inspected the occurrence of IPV among middle-aged adults. Focusing on the investigation of the associations between ADHD and IPV among young people while neglecting these dynamics in middle-aged adults could lead to inaccurate conclusions when taking into account that the highest perpetration and victimization rates were documented for adults ranging from 30 to 40 years old^24^. Hence, more research is needed to disentangle to what extent IPV occurs in romantic relationships involving women or men with ADHD at different ages. Prior research has validated a reduction of intimacy levels and lower marital satisfaction within intimate relationships of middle-aged ADHD patients compared to those of individuals without ADHD^37–39^.

Basically, women seem to be more frequently victims and men more frequently offenders of IPV. Although it seems plausible that being a perpetrator or victim of IPV might depend on gender also in the context of ADHD, only few studies integrated a differentiated gender analysis when focusing on ADHD and IPV. For example, Wymbs et al.^34,36^ found that women, regardless of ADHD status, were more likely than men to become victims of IPV, although no differences emerged in the context of perpetration. In another study, however, females with childhood and persistent ADHD experienced more physical IPV compared to females with low persistent ADHD or those never diagnosed^33^. Moreover, little is known about the impact of ADHD on the risk of women to become IPV perpetrators. The given evidence also raises questions about the effect of gender in the link between ADHD and IPV as to whether men with ADHD tend to behave more aggressively toward their partners and rather become perpetrators than men without ADHD^40^. It seems reasonable to suggest that if ADHD had an effect on perpetration and victimization, then women with ADHD would be more likely to show violence and men more likely to become victims compared to women and men without ADHD.

The present study aimed at extending prior research on the associations of ADHD with IPV in a large sample of 18–65-year-old male and female adults, considering specific characteristics of IPV, such as violence direction (perpetration and victimization) and violence form (psychological and physical).

We hypothesized that adults with ADHD would report greater rates of IPV perpetration and victimization than those without ADHD. We also assumed that in general, men would show higher perpetration rates, whereas women would rather report victimization. However, both IPV perpetration and victimization rates were expected to assimilate between genders when ADHD was present.

## Methods

### Procedures

Data was collected anonymously via the online survey software Unipark between 5 December 2021 and 21 March 2022. The questionnaire asked for socio-demographic data (age and gender), prior ADHD diagnosis, and other psychiatric diagnoses. Moreover, current ADHD symptoms as well as experiences of IPV victimization and perpetration were assessed. Study participation was voluntary and unpaid after giving informed consent in accordance with the Declaration of Helsinki. The study was approved by the Ethics Committee of the Saarland Medical Association (312/21, approval date 1st March 2021).

### Participants

Participants were recruited in different ways. Former adult patients from the ADHD outpatient consultation department of the Institute for Forensic Psychology and Psychiatry, Homburg, Germany, were contacted via mail or telephone and asked whether they would take part in the survey. Additionally, the link to the survey appeared on the website of Germany´s most prominent self-help organization, (www.adhs-deutschland.de) as well as in various ADHD support groups on Facebook. Study flyers became also noticeable at the psychotherapeutic clinic of the University of Trier, and further invitations were sent through respective e-mail distribution lists of the University of Trier. Likewise, healthy (non-ADHD) participants were addressed through flyers and an e-mail distribution list of the University of Trier, as well as through social media websites.

### Measurements

#### Wender-Reimherr self-report questionnaire on adult ADHD

The Wender-Reimherr self-report questionnaire on adult ADHD is the shortened version of the Wender-Reimherr Adult Attention Deficit Disorder Scale, also known as WRAADDS^41^, to assess adult ADHD symptomatology ecologically based on the Utah criteria of adult ADHD. The German version of the Wender-Reimherr self-report questionnaire, also known as WR-SB^42^, contains 53 items measuring ten symptom domains and six additional items that address three further problem areas but are not assigned to any of the categories mentioned and, thus, not included in the evaluation. For an ADHD diagnosis, both the criteria Inattention and Hyperactivity must be met in combination with at least two of the following: Temperament, Affective Lability, Emotional Overreacting, Disorganization, or Impulsivity. Items are answered on a five point Likert scale ranging from 0 (= strongly disagree) to 4 (= strongly agree). The German WR-SB showed an adequate discriminatory power and a high internal consistency for the total scale (α = 0.978) as well as for individual subscales (α between 0.805 and 0.978^43^). Table 1 displays the internal consistency values of each WR-SB scale in the present sample.

**Table 1:** Cronbach’s Alpha for individual WR-SB subscales.

Criterion	Number of items	Cronbach’s α
Inattention	6	0.932
Hyperactivity	3	0.805
Temperament	3	0.899
Affective lability	4	0.847
Emotional overreacting	4	0.896
Disorganization	6	0.923
Impulsivity	5	0.861
Oppositional symptoms	9	0.883
Academic problems	4	0.790
Social attitude	9	0.853
Total scale	53	0.978

*N* = 316.

#### Conflict Tactics Scale 2

The Conflict Tactics Scale 2, also known as CTS 2^44^, is a widespread instrument used to measure intimate partner violence in marital, cohabiting, or dating relationships. The present study used the German translation of the CTS 2^45^. A total of 26 questions were presented in pairs to obtain data on the behavior of both partners, also referred to as the victim and the offender perspective, assigned to the three subscales of psychological aggression (8 items), physical assaults (12 items), and use of negotiation (6 items). Other than the original version, the German CTS 2 excludes any sexual coercion and injury scales^45,46^. The psychological aggression subscale assesses both verbal and non-verbal emotional actions. In contrast, the physical assaults subscale captures various forms of physical abuse, such as pushing, the use of firearms, strangulation, kicking, etc. Use of negotiation refers to cognitive and emotional strategies for conflict resolution, precisely the extent to which positive affect is communicated. Since the present study focused on the amount of psychological and physical perpetration and victimization rather than positive affect, the use of negotiation subscale was neglected. The frequency of respective behavior or experiences were reported on a five-point Likert scale ranging from 1 (= this has never happened) to 5 (= this has happened very often). We focused on examining the psychological aggression perpetration (α = 0.842) and victimization (α = 0.837) scores as well as the physical assault perpetration (α = 0.751) and victimization (α = 0.805) scores^44^.

#### Covariates

In addition to the abovementioned variables of interest, self-reported age as well as gender identification was assessed.

### Statistical analysis

Data were processed in IBM SPSS version 29.0. To investigate the associations between ADHD and IPV, we first conducted a MANOVA with post hoc Holm-Bonferroni corrections. Psychological and physical violence scales, each from perpetrator and victim perspective, served as dependent and ADHD versus no-ADHD subgrouping as independent variables. Second, we performed similar analyses to investigate differences between males and females.

Partial eta-squared (η_p_^2^) was used as effect size with values of 0.01, 0.06, and 0.14 representing small, medium, and large effects, respectively^47^. The general level of significance was set a *p* ≤ 0.05.

To further examine the predictive effects of ADHD on IPV, we performed multiple linear regression analyses. First, we included gender and age as independent and the IPV subscale scores as dependent variables. Second, we tested remaining predictive effects when further independent variables such as ADHD and the interaction of age and gender were considered.

## Results

Of 372 participants who initially took part in the study, 49 (13%) participants did not describe any current ADHD symptoms despite reporting a previous ADHD diagnosis. In order to clearly differentiate between those with and without ADHD history, those participants had to be excluded. Moreover, 4 (1%) participants under 18 years were excluded. Additionally, 3 participants who had identified their gender as diverse had to be excluded because this gender-related subsample was too small for adequate statistical analysis. Finally, 316 participants aged between 18 and 68 years (M = 35.91, SD = 14.16 years), of those 220 female and 96 male, were included. Based on their self-reports in the WR-SB, 131 (41.5%) participants met the diagnostic criteria for adult ADHD. 114 (87.0%) of them indicated a previous ADHD diagnosis, thus representing the ADHD group. The control group contained 185 (58.5%) participants neither diagnosed with ADHD in the past nor fulfilling the diagnostic threshold for current ADHD, according to the WR-SB. As reported by the participants themselves, 7.0% (n = 22, min: 4, max: 17) had received an ADHD diagnosis prior to reaching the age of 18. In contrast, 28.8% (n = 91, min: 18, max: 64) were diagnosed with ADHD after attaining adulthood.

In the overall sample, 27.5% (n = 87) reported the presence of an additional mental health disorder apart from ADHD. In our study, depression (n = 45, 14.2%) emerged as the most frequently reported comorbidity, followed by anxiety disorders (n = 13, 4.1%), post-traumatic stress disorder (n = 10, 3.2%), borderline personality disorder (n = 6, 1.9%) and obsessive–compulsive disorders (n = 4, 1.3%). In the ADHD group, 49.3% acknowledged an additional clinical diagnosis, whereas only 13.5% in the control group reported any mental health disorder.

Gender distribution related to ADHD was not balanced (Chi^2^(1) = 12.06, *p* < 0.001) with females being over-represented among those with ADHD (81.6%, adjusted residual = 3.5).

Table 2 presents differences between ADHD and non-ADHD participants on the IPV subscales. Adults with ADHD showed higher scores on each of the IPV perpetration and victimization subscales than those without ADHD.

**Table 2:** Mean differences of ADHD and non-ADHD participants on psychological aggression perpetration, psychological aggression victimization, physical assault perpetration, and physical assault victimization (F values).

IPV subscale	M (SD)			
	Non-ADHD (n = 202) (*n* = 185)	ADHD (*n* = 114)	*F* (1315)	*η*_p_^2^
Perpetration				
Psychological	12.63 (3.39)	17.11 (6.74)	61.92***	0.165
Physical	12.61 (1.47)	14.18 (3.28)	34.14***	0.098
Victimization				
Psychological	12.32 (3.64)	15.54 (6.15)	34.02***	0.098
Physical	12.68 (1.92)	13.71 (3.02)	13.62***	0.042

*η*_p_^2^ < 0.01 and ≤ 0.06 = medium, *η*_p_^2^ ≥ 0.14 = large. ***p ≤ 0.001.

Table 3 shows the differences between female and male participants on the IPV subscales. Whereas no significant differences emerged with regard to physical perpetration or victimization, females showed higher scores than men on both psychological perpetration and victimization.

**Table 3:** Mean differences of gender identification (female and male participants) on psychological aggression perpetration, psychological aggression victimization, physical assault perpetration, and physical assault victimization (F values).

Gender	M (SD)			
	Female (n = 220) (*n* = 185)	Male (*n* = 96)	*F* (1315)	*η*_p_^2^
Perpetration				
Psychological	14.72 (5.61)	13.17 (4.40)	5.78*	0.018
Physical	13.33 (2.64)	12.82 (1.70)	2.96 n.s	0.009
Victimization				
Psychological	13.93 (5.23)	12.46 (4.05)	6.00*	0.019
Physical	13.20 (2.63)	12.73 (1.83)	2.49 n.s	0.008

n.s. = not significant; *η*_p_^2^ < 0.01 and ≤ 0.06 = medium, *η*_p_^2^ ≥ 0.14 = large. *p ≤ 0.05.

Irrespective of age, gender proved to be a significant predictor for psychological aggression perpetration and victimization (Tables 4, Model a), yet only when ADHD was not included in the model. ADHD positively predicted both physical and psychological perpetration and victimization under consideration of age and gender (Table 4, Model b). However, no meaningful interactions between ADHD and gender were found (Table 4, Model c).

**Table 4:** Associations between ADHD, gender, age, and IPV.

		Δ*R*^2^	*p*	Predictor	*B*	95%CI	β	*p*
Psychological aggression perpetration	Model a	0.02	0.040	Gender	− 1.55	− 2.82, − 0.28	− 0.13	0.017
				Age	0.02	− 0.02, 0.06	0.05	0.389
	Model b	0.15	< 0.001	Gender	− 0.65	− 1.85, 0.54	− 0.06	0.283
				Age	0.02	− 0.02, 0.06	0.06	0.226
				ADHD	4.39	3.24, 5.53	0.40	< 0.001
	Model c	0.00	0.621	ADHD * gender	0.68	− 2.02, 3.38	0.03	0.621
Physical assault perpetration	Model a	0.01	0.190	Gender	− 0.51	− 1.08, 0.07	− 0.10	0.086
				Age	− 0.01	− 0.03, 0.01	− 0.4	0.534
	Model b	0.09	< 0.001	Gender	− 0.19	− 0.76, 0.37	− 0.04	0.499
				Age	− 0.00	− 0.02, 0.01	− 0.02	0.655
				ADHD	1.53	0.99, 2.07	0.31	< 0.001
	Model c	0.00	0.934	ADHD * gender	0.05	− 1.22, 1.33	0.01	0.934
Psychological aggression victimization	Model a	0.03	0.011	Gender	− 1.46	− 2.26, − 0.29	− 0.14	0.015
				Age	0.03	− 0.00, 0.07	0.10	0.078
	Model b	0.09	< 0.001	Gender	− 0.83	− 1.98, 0.32	− 0.08	0.155
				Age	0.04	0.00, 0.08	0.11	0.041
				ADHD	3.10	2.00, 4.20	0.30	< 0.001
	Model c	0.00	0.744	ADHD * gender	− 0.43	− 3.03, 2.17	− 0.02	0.744
Physical assault victimization	Model a	0.01	0.260	Gender	− 0.47	− 1.05, 0.12	− 0.09	0.117
				Age	0.01	− 0.01, 0.02	0.03	0.637
	Model b	0.04	< 0.001	Gender	− 0.27	− 0.85, 0.32	− 0.05	0.373
				Age	0.01	− 0.01, 0.02	0.03	0.545
				ADHD	0.98	0.42, 1.54	0.20	< 0.001
	Model c	0.00	0.674	ADHD * gender	0.28	− 1.04, 1.6	0.03	0.674

Model c includes all mentioned variables, only the interaction term is displayed; female was coded as 0 and male was coded as 1.

## Discussion

The current study aimed at clarifying the relationship between ADHD and IPV in an age-diverse sample of adults. Significant differences were found in psychological and physical IPV between individuals at risk for ADHD and those without. Adults at risk for ADHD were more frequently both victims and perpetrators of IPV irrespective of gender and age. These results confirmed our hypotheses and show that adults at risk for ADHD are of elevated risk for both IPV perpetration and victimization when comparing them to adults without any ADHD history^33,34^. Regarding the aim of this study, the focus was on current severity of ADHD symptoms and their immediate impact on intimate partner violence. Therefore, individuals with a history of ADHD diagnosis who had minimal current symptoms were excluded.

In accordance with previous research findings, females in our sample were more frequently victims, but also perpetrators of psychological IPV than males^48^. Yet, studies on gender-specific aggression in general have stated that females rather tend toward non-physical than physical aggression compared to males^49^. On the other hand, unlike previous research^34,36^, the current study did not uncover any significant gender-specific differences between physical assault perpetration or victimization. It can be assumed that our findings underscore the potential for reciprocal physical violence, where both individuals in the relationship are engaging in violent behavior towards each other^50^. It is important to recognize that individuals who experience IPV may resort to perpetrating IPV as a form of self-defence^51^. Moreover, commonly reported differences in aggression between females and males may not have been found in our sample since ADHD turned out to be a leading influence on IPV rather than gender itself.

Nevertheless, the outcomes of the present study show that presumed gender disparities in IPV may be less pronounced than previously thought, at least in the context of ADHD. The issue of men experiencing IPV still seems to remain a taboo topic. Research indicates that men who are victims of IPV are less inclined to seek assistance for their victimization compared to women^52^. However, there has been an increase in assaults on men by their female partners^53^. Despite commonly prevalent stereotypes that consider men as invulnerable and physically superior to women, who, on the other hand, are assigned the role of victims only, women may also exhibit aggression towards men, with a particular emphasis on the perpetration of psychological violence against their partners^54^. However, due to the societal perceptions of masculinity and femininity, instances of male victimization and female-perpetrated violence rarely receive public attention, leading to the current limited state of research on this matter^55^.

The given results have to be considered in the light of both strengths and limitations. One major advantage compared to most previous studies on ADHD and IPV was the survey of a somewhat older and more age-diverse sample that ensured higher levels of representativeness, especially when taking into account that the online survey reached a broader target population than would have been possible by on-site examination^30,31,33^. Moreover, the use of empirically sound assessment instruments that included a multidimensional view on both ADHD and IPV allowed a more sophisticated perspective on these constructs and their dynamics. It should be noted, however, that the WR-SB is only one of several tools that are required in the diagnostic process for assessing adult ADHD symptomatology. The WR-SB, in isolation, is not sufficient to establish an ADHD diagnosis. Especially individual impairment must not be neglected when diagnosing ADHD, which was, however, beyond the scope of the present study. Yet, the question arises whether specific dimensions of ADHD like impulsivity could have played a significant role in the association between ADHD and the different IPV forms which should be examined in future studies. Arrondo et al. already identified hyperactive, impulsive, and inattention symptoms as risk factors for adult IPV^56^. Impulsivity and poor self-control can increase the probability of violent acts^57^. Inattentiveness might raise the risk of becoming a victim in general^58^.

Furthermore, social desirability may have influenced self-reports^59^. Although using an online survey could have enhanced the perception of anonymity compared to completing the questionnaires on-site and, thus, may have reduced respective bias, online questionnaires can lead to lower internal validity, as confounding variables (especially the presence of a partner) are more difficult to control. Even though completing the 10–15-minute online survey could be interrupted and continued at a later time if disruptions occurred, inattentiveness as a key symptom of ADHD could have contributed to higher dropout rates in those with limited ability to concentrate. No attention-check questions or other means of identifying incapable participants or bots were used in this study. Moreover, the disproportion with regard to male and female gender in our sample may have introduced a potential bias. It is also important to highlight that during the survey period, there were temporary restrictions on public life due to COVID-19. Numerous nations have noted an increase in instances of domestic violence following the enforcement of COVID-19-induced lockdowns and measures promoting physical distancing^60^. Eventually, it cannot be excluded that symptoms of comorbid mental health problems apart from ADHD might have contributed to IPV as well. Although we had asked participants whether they had received any psychiatric diagnosis before, we did not control for comorbidity in the present study because it was only assessed by a single self-report item that did prevent a sophisticated diagnostic evaluation of respective symptoms. Future studies should take a closer look into the dynamics among ADHD, IPV and comorbid psychiatric disorders based on clinician-administered assessment.

## Conclusions

ADHD can pose significant challenges for those affected and their partners, as symptoms can foster severe relationship conflicts in terms of disagreements, dissatisfaction, mistrust, accusations, and unmet expectations, eventually increasing the risk of IPV. Taking into account that ADHD is a common mental disorder in adulthood, this research area deserves further attention. The present study highlights the importance of prevention as well as psychotherapeutic and pharmacological intervention for ADHD patients not only in the light of self-directed burden but also interpersonal problem behavior. However, the prerequisite would be a sufficiently accurate diagnosis. Remarkably, 13.0% of the participants of the present study who met the criteria for ADHD according to WR-SB had never received a formal ADHD diagnosis. However, it should be noted that the diagnosis of ADHD is particularly intricate and time-consuming. Therefore, a valid diagnosis cannot be established through a self-report questionnaire but requires an adequate clinical examination.

Effective treatment would benefit those affected with ADHD and assist their fellow human beings, potentially preventing IPV. While medication has been recommended as first-line treatment for ADHD in adults, psychoeducational elements that stress the issue of IPV should also be an integral part of additional psychotherapeutic approaches. It is imperative to sensitize individuals with ADHD, but also their partners and the community, to the issue of IPV and provide them with suitable strategies and skills. Finally, research and practice must not be blinded by common gender-associated stereotypes in the context of IPV but should implement sensitive ways to approach women and men as both victims and perpetrators of IPV.

## Data Availability

The data presented in this study are available on request from the first author (J.M.).
